# Effect of Reactive Gas Composition and Duty Cycle in Low-Pressure DC-Pulsed Plasmas on the Surface Oxidation of Nitrile Rubber

**DOI:** 10.1021/acsomega.6c05228

**Published:** 2026-08-21

**Authors:** Mateus S. Zanzi, Gabriel B. Dutra, Diego A. Duarte, Kleber V. Paiva, Guilherme M. O. Barra

**Affiliations:** † Graduate Program in Material Science and Engineering (PGMAT), Federal University of Santa Catarina, Florianópolis, Santa Catarina 88040-900, Brazil; ‡ Graduate Program in Mechanical Science and Engineering (PPGECM), 28117 Federal University of Santa Catarina, Joinville, Santa Catarina 89219-600, Brazil; § Graduate Program in Physics (PPGF), Santa Catarina State University, Joinville, Santa Catarina 89219-710, Brazil

## Abstract

Nitrile rubber (NBR) is widely used in components across several industrial sectors due to its properties that are suitable for engineering applications. Surface-environment interactions often limit performance, which depends critically on surface characteristics, especially barrier properties for sealing and membrane applications. This study investigates the effects of low-pressure direct-current pulsed plasma treatment in O_2_/Ar atmospheres on NBR surfaces to generate oxidized layers. Samples were treated at duty cycles of 30% and 70% with oxygen contents of 20% (low) and 80% (high). Nanoindentation post-treatment revealed increased surface contact stiffness, while differential scanning calorimetry (DSC) confirmed unchanged bulk properties. Attenuated total reflectance Fourier transform infrared (ATR-FTIR) spectroscopy indicated the incorporation of oxidized groups and reduced C–H signals. The highest duty cycles (70%) improved sputtering species production, and the highest oxygen content (80%) enhanced O ion creation and surface oxidation, as evidenced by optical emission spectroscopy (OES) and X-ray photoelectron spectroscopy (XPS). XPS depth profiling showed a rising oxidation profile (oxygen implantation) at the treated surface. Based on a plasma-surface interaction mechanism, the condition with 80% oxygen and 30% duty cycle yields the highest surface oxidation level. It potentially enhances barrier properties, contributing to plasma-parameter optimization for tailored NBR surface modifications.

## Graphical abstract

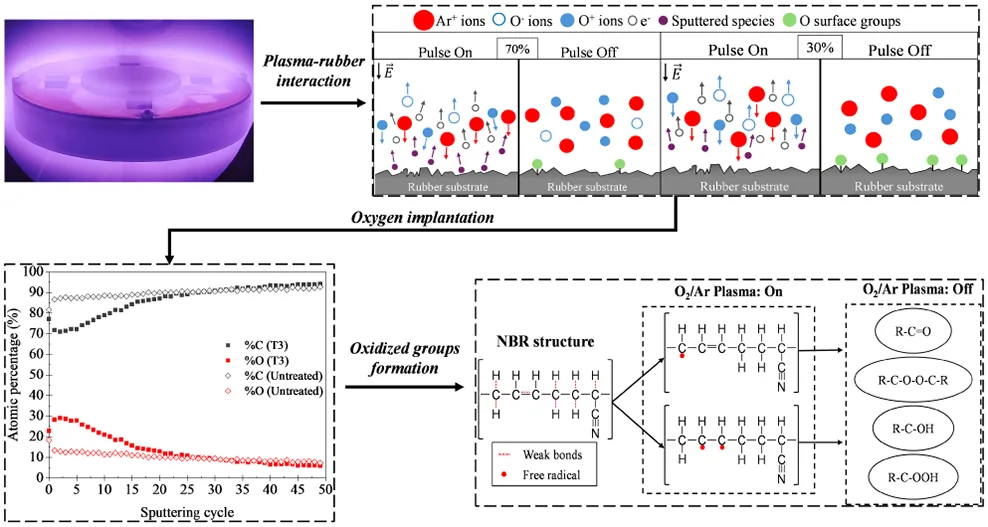

## 1 Introduction

Rubbers constitute a group of polymers developed to meet specific engineering requirements and to provide particular properties.^1^ Rubber applications are generally associated with energy generation,^2^ transport and storage of gas,^3^ damping,^4^ and sealing elements for extreme environmental conditions.^5–7^ However, for some applications, changes in specific rubber properties may be necessary, particularly in membrane applications, where surface and bulk properties can influence component performance, selectivity, fouling behavior, and hydrophobic recovery.^8^ Moreover, these treatments can be used to improve rubber performance and extend the service life of these materials.

Typical rubber modifications are associated with enhanced adhesion,^9–12^ superhydrophobic coatings,^13,14^ and improved aging resistance.^15–18^ Surface modifications are mainly carried out by using chemical deposition and plasma treatments to modify the rubber surface. Plasma surface modification is a well-established and controlled method for modifying the surface of these materials.^19–22^ Plasma treatments of polymers cause the formation of functional groups, tailoring the surface properties of polymers and polymer composites.^23,24^ Specifically, low-pressure direct-current pulsed plasmas (DC-pulsed plasmas) play an important role in polymer treatments, as these plasmas allow us to control the plasma atmosphere, heating, and surface reactions (fragmentation, polymerization, oxidation, etc.) during the voltage pulses, providing homogeneous results for applications.^25,26^

The major plasma components, including high-energy and/or ionized particles, generate free radicals on the polymer surface and can induce the formation of organic compounds, chain cross-links, and volatile products, such as hydrogen, within the near-surface region of the polymer surface (50 Å to 10 μm), while bulk properties remain unaffected.^26^ The proper atmosphere selection for plasma treatment is responsible for the characteristic groups formed on the polymer surface.^27^ By combining an inert gas (*e*.*g*., Ar, He) with a reactive gas (*e*.*g*., O, N, and F), surface functionality can be increased due to enhanced radical formation and subsequent surface reactions. Inert gases, together with UV irradiation, create free radicals, whereas reactive gases interact with active sites on the polymer surface, leading to the formation of functional groups, such as carboxyl, hydroxyl, and amide groups.^8,26,28,29^

The most common functionalities introduced onto rubber surfaces by oxygen plasma are carbon–oxygen groups such as C–O, C=O, and O–C=O.^9,10,19,30,31^ Nonetheless, other important structural changes may occur when active sites interact with oxygen species, such as chain scission and cross-linking between rubber chains. Chain scission reduces the molecular weight and increases the chain mobility.^32^ The balance between these reactions depends directly on the rubber material and the plasma treatment conditions.^25^

Rubber surface modifications by plasma-assisted processes generate radicals that can either react with functional groups through chemical deposition^33,34^ or induce surface changes by physical bombardment followed by chemical reactions with plasma-atmosphere gases.^19^ Bielinski et al. evaluated different plasma atmospheres, including He, O, and Ar, for different rubber materials in terms of friction and wettability after being submitted to high-voltage ion bombardment.^35^ The authors showed that cross-linking and surface oxidation took place by hydrogen release. Maia et al. evaluated ethylene propylene diene monomer (EPDM) rubbers treated with afterglow microwave plasma under different gas compositions and treatment times, resulting in the incorporation of oxygen- and nitrogen-containing groups onto the rubber surface.^36^ Shen et al. showed that changes in the tribological properties of NBR after plasma treatments were closely related to modifications in surface microstructure and roughness, as well as the incorporation of oxygen-containing functional groups.^16^

Despite numerous experimental efforts to modify rubber surfaces via plasma treatment, a comprehensive evaluation of the effects of the processing parameters remains unclear.^9,10,16,37–39^ To address this research gap, this study aims to modify the surface of NBR via low-pressure DC-pulsed plasma treatments to enhance barrier properties by forming an oxidized surface layer without altering the bulk characteristics. Plasma treatments with different Ar/O_2_ mixtures and varying pulse-on durations of the plasma power source in pulse mode were carried out to induce surface oxidation on the NBR samples. Nanoindentation tests assessed the effects on the mechanical properties, while differential scanning calorimetry (DSC) was used to evaluate the rubber bulk properties after plasma exposure. Plasma emissions were examined using optical emission spectroscopy (OES) analysis. The chemical composition and characterization of the samples were analyzed using attenuated total reflectance Fourier transform infrared (ATR-FTIR) and X-ray photoelectron spectroscopy (XPS) with depth-profile analyses. A proposed mechanism is presented to elucidate the plasma-rubber interaction and chemical reactions involved in the different treatments.

## 2 Experimental Section

### 2.1 Materials

In this study, a nitrile rubber (NBR) containing 33 wt % acrylonitrile (ACN) was used. The compound formulation contained 40 phr (parts per hundred rubber) carbon black, 1.6 phr zinc oxide, and no plasticizer. A protective agent (2 phr), consisting of paraphenylenediamine (P-PDA) and 3-methylquinoline (TMQ), was incorporated to enhance the durability and service life of the vulcanizate. The curing system (3 phr) included a sulfur donor, tetramethylthiuram disulfide (TMTD), *N*-cyclohexyl-2-benzothiazole sulfenamide (CBS), and 4,4′-dithiodimorpholine (DTDM). Stearic acid was added as a processing aid at 4.4 phr. The rubber samples were manufactured as compression-molded sheets measuring 200 × 200 mm^2^ with a thickness of 2 mm. The specimens were cut into 10 × 10 mm^2^ squares for treatment and characterization.

The samples were manually washed with a neutral detergent solution prepared with distilled water at a detergent-to-water ratio of 1/10. To remove residual oil and waxes, the samples were cleaned in an ultrasonic bath (40 kHz) at room temperature for 2 min. Afterward, all samples were dried under an N_2_ flow to remove possible surface contamination.^16^ The cleaned samples were stored in a glass desiccator filled with silica gel at room temperature.

Because of the specific optical and physical properties of the material, AFM roughness measurements could not be performed with the available instrumentation. As an alternative approach, the surface roughness of the nitrile rubber was evaluated by using a contact profilometer (Mitutoyo SJ-210). The sample roughness was determined as the mean of the average roughness (*R*_a_) values obtained from at least three measurements of each test piece.

### 2.2 Plasma Treatment

Surface modifications were performed using low-pressure DC-pulsed plasma in a 21-L stainless-steel vacuum chamber, as illustrated in Figure 1.

**Figure 1:**
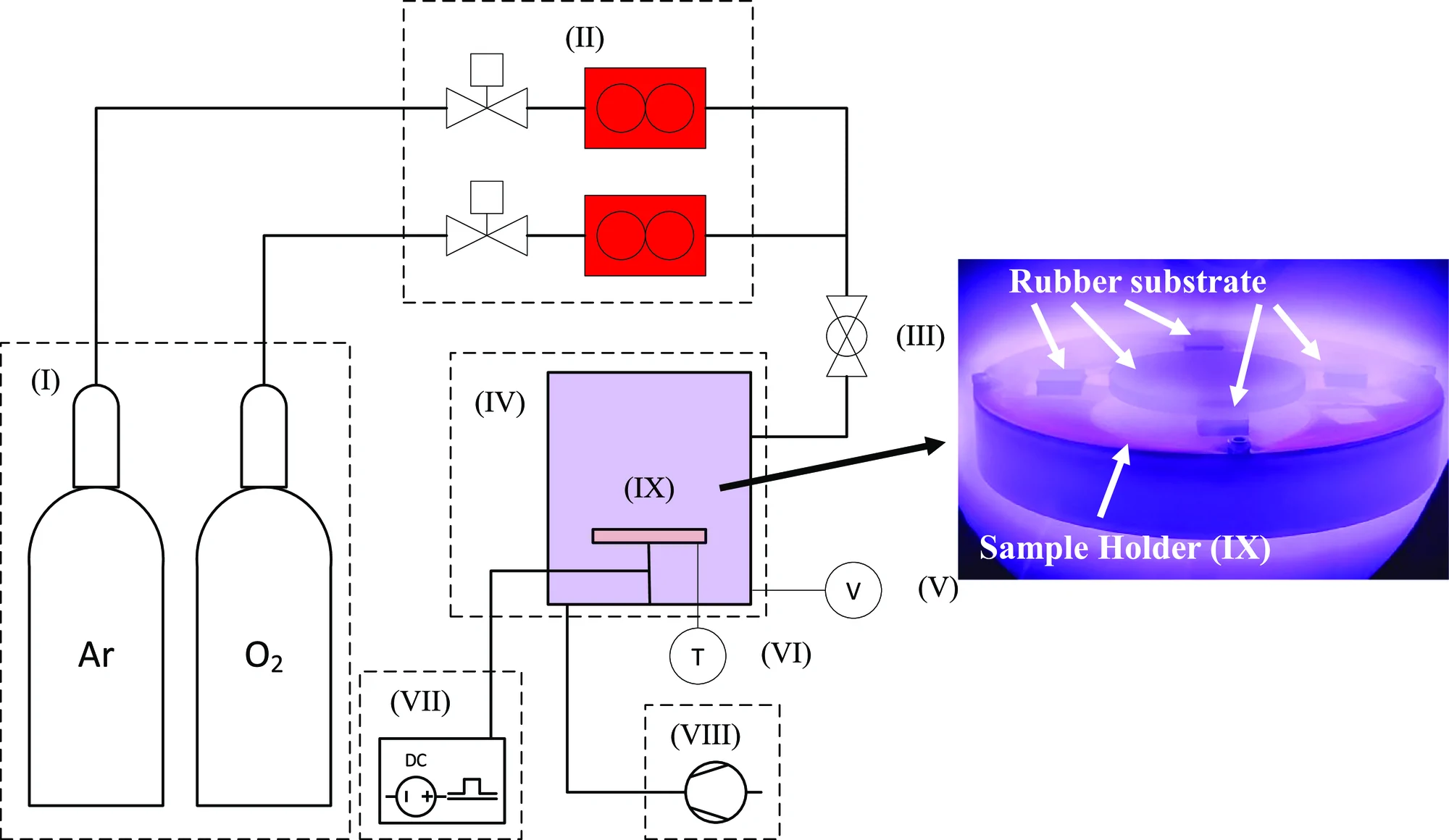
Experimental setup: I—gas cylinder; II—mass flow controller (MFC); III—ball valve; IV—vacuum chamber; V—vacuum gauge; VI—K-type thermocouple; VII—power Source; VIII—mechanical pump; IX—copper substrate holder.

Plasma treatment with polymers should be well controlled to avoid high electron temperatures and polymer degradation.^26,40^ Low-energy levels, approximately 100 W, may be sufficient to enhance the formation of functional groups on the polymer surface.^26^ The DC power source (DCMP 1500, T&C Power Conversion Inc.) used in this work operates in continuous mode (up to 1500 W) or in DC-pulsed mode, up to 1050 W. The pulsed discharges were performed at 120 kHz with 100 W for 10 min. Considering that each complete cycle of a pulsed discharge comprises pulse-on and pulse-off stages, the duty cycle is defined as the percentage of pulse-on duration relative to the total cycle duration, and it can influence the surface composition during plasma surface modification.^20^ Based on this, two duty-cycle levels were employed, 30% and 70%, for which the pulse-on durations were 2.50 and 5.83 ms, respectively.

Energy efficiency can be improved, and gas-phase recombination processes are much slower under low-pressure conditions.^20^ The pressure was maintained using a two-stage rotary vane pump (GVD5, Atlas Copco), measured with a Pirani gauge (TPR 270, Pfeiffer), and monitored by a reading unit (OmniControl 200, Pfeiffer). The adopted working pressure was 250 mTorr (∼0.33 mbar), since the base pressure was around 30 mTorr (∼0.04 mbar).

The focus of this work was on improving the oxygen content of the rubber surface. With the aid of a mass flow controller (MFC; SEC-E40, Horiba Stec) controlled by a hub (Power hub basic, Horiba), two environments were established with a high-purity gas mixture containing molecular oxygen (99.99% O_2_) and argon (99.99% Ar) for the plasma treatments, as argon ions can enhance oxygen functionalities.^28,29^

The atmospheres created consisted of mixtures with proportions of 20%/80% (O_2_/Ar) and 80%/20% (O_2_/Ar). The sample identification for the respective plasma treatments is presented in Table 1.

**Table 1:** Sample Identification for the Untreated and Plasma Treated Samples

	Plasma parameters				
Sample Id	Duty cycle	O_2_ content	Ar content	O_2_ partial pressure (mTorr)	Ar partial pressure (mTorr)
Untreated	-	-	-	-	-
T1	30%	20%	80%	50.0	200.0
T2	30%	80%	20%	200.0	50.0
T3	70%	20%	80%	50.0	200.0
T4	70%	80%	20%	200.0	50.0

The temperature increase of the substrate holder from ambient temperature (∼25 °C) during plasma treatment was 54 and 75 °C for treatments performed at 30% and 70% duty cycles, respectively, because the longer pulse-on duration at 70% duty cycle promotes a higher heating effect. This temperature increase and treatment time are not expected to affect the NBR properties.^41,42^ The initial source voltage and current for treatments with a 70% duty cycle were 567 V and 213 mA, respectively; for treatments with a 30% duty cycle, the initial source voltage and current were 624 V and 290 mA, respectively. These parameters are automatically adjusted by the internal control system of the power source to maintain a constant RMS plasma power. This regulation follows the relationship $P_{\mathrm{RMS}}=V\times I\times \sqrt{DC}$ , where *P_RMS_* is the RMS power, *V* is the peak voltage, and *I* is the peak current. Due to the Joule effect, the subsequent increase in temperature and electrical resistance requires continuous adjustment of the source parameters to strictly maintain the adopted plasma power of 100 W.

### 2.3 Nanoindentation

The surface contact stiffness was evaluated by nanoindentation tests to capture the mechanical response of the modified outermost layer (operating on a micrometer scale) without significant influence from the underlying bulk stiffness. In this context, nanoindentation measurements were performed using a nanoindentation system (CETR) equipped with a Berkovich diamond probe. The applied load ranges from a few μN to 200 mN, while the penetration ranges from a few nm to a few μm.^43^ The indentation tests consisted of applying six progressive load steps up to the maximum test load (50 mN) in nine points along the specimen surface, which form a 3 × 3 square matrix of points.

During the tests, the applied force (mN) and penetration depth (μm) were measured continuously. The contact stiffness was estimated in the UMT software using the Oliver–Pharr method, which is based on the slope of the unloading regime of the force–displacement curve (the elastic contact stiffness) for each load step.^43,44^ The contact stiffness in each evaluated condition comprises the average of 18 points, where each sample is composed of two specimens.

### 2.4 Differential Scanning Calorimetry (DSC)

In elastomeric materials, macromolecular chain mobility is directly related to the energy storage capacity of the matrix, which dictates the glass transition temperature (*T*_g_). Cross-linked polymer networks exhibit restricted chain mobility, which typically correlates with alterations in thermal transitions. In this work, the *T*_g_ of the rubber surfaces was determined using differential scanning calorimetry (DSC). The tests were performed in an inert atmosphere (N_2_) using the DSC25 (TA Instruments) equipment. The specimens had masses ranging from 5 to 10 mg and were subjected to thermal cycles from −40 to 30 °C at a heating rate of 10 °C/min. All samples underwent conditioning cycles to remove manufacturing-induced thermal memory. *T*_g_ was determined using TA analyzer software, and the results are reported as the average of at least three samples per condition.

### 2.5 Attenuated Total Reflectance–Fourier Transform Infrared (ATR-FTIR) Spectroscopy

To assess the functional groups in all samples, chemical analysis was performed using Fourier transform infrared region spectroscopy (FTIR), with a PerkinElmer infrared spectrometer (Frontier model) equipped with an attenuated total reflection (ATR) accessory using a diamond/ZnSe crystal. The analysis range was 4000–600 cm^–1^, and 32 scans were applied per sample. All ATR-FTIR analyses were performed in the surface region of the specimen volume.

### 2.6 Optical Emission Spectroscopy (OES)

Optical emission spectroscopy (OES) analyses were used to identify and record plasma emissions during different plasma treatments. These spectra were obtained from 250 to 1050 nm range using a compact spectrometer (OceanOptics, SR4). The emitted radiation, collected via an optical fiber (solarization-resistant fiber) with a 600 μm diameter and an UV/vis collimating lens (200–2500 nm), was transmitted to the spectrometer and then to the acquisition software (OceanView). The spectral results comprise the average of at least 10 acquisitions with a 100 ms integration time.

### 2.7 X-ray Photoelectron Spectroscopy (XPS)

In this study, X-ray photoelectron spectroscopy (XPS) analyses were performed to quantify the atomic concentrations of oxygen and carbon in the samples and to determine their oxidation states. These analyses were conducted using a Thermo Scientific K-Alpha spectrometer with monochromatic Al–K_α_ radiation (1486.6 eV) under a base pressure of 10^–8^ mbar. Wide-scan spectra were evaluated between 0 and 1100 eV and comprised 10 scans with a pass energy of 200.0 eV, an energy step of 1 eV, and a dwell time of 0.01 s, while high-resolution spectra (O 1s and C 1s) comprised 10 scans with a pass energy of 20 eV, an energy step of 0.1 eV, and a dwell time of 0.1 s. The flood gun was used to compensate for charging effects.

To investigate the oxygen concentration across the sample thickness (from the externally treated surface to the inner region), depth-profile analyses were conducted using a 1000 eV argon-ion gun. XPS profiles were recorded over 50 cycles, each with a 5-s etching time at a spot size of 400 μm.

The spectral analyses were carried out using Khervefitting software v1.8.^45^ The main peak in the C 1s core level, associated with aliphatic carbon (C–C), was used to correct the binding energy scale across all samples, with 284.8 eV as the reference.^46^ High-resolution spectra were fitted using a Gaussian–Lorentzian (area) model with least-squares as the fitting method, where the Shirley method was applied for background subtraction. The peak fitting in the C 1s core level employed a set of full width at half-maximum (FWHM) and Lorentzian/Gaussian proportion (*L*/*G*) in the product model. The FWHM values used for deconvolution of C 1s core-level peaks (1 - C–C/C–H; 2 - C–O/C–N/C–OH; 3 - C=O; and 4 - O–C=O) were 1.2–2.0, 1.2–2.0, 1.2–2.2, and 1.2–2.2, while the *L*/*G* proportion ranged from 55 to 70% for peaks 1 and 2, and 30 to 50% for peaks 3 and 4. The fitting of the O 1s high-resolution core-level regions considered only a main peak, with FWHM of 1.2–2.0 and an *L*/*G* proportion of 50% to 70%. The use of these fit parameters ensured a low relative standard deviation (RSD < 2%) and a high coefficient of determination (*R* ^2^ > 0.99).

The concentrations of C 1s and O 1s core levels were calculated from the proportions of the integrated areas. The respective areas were calculated by the relation *S*_*i*_ = *A*_*i*_/*RSF*_*i*_, where *S*_*i*_ is the corrected area concerning the area obtained from the spectra integration (*A*_*i*_) and the relative sensitivity factor (*RSF*_*i*_) for the respective core-level element (_*i*_). Thus, the concentrations for oxygen [O] and carbon [C] (in percentage) were defined by [O] = 100 × *S*_O_/(*S*_O_ + *S*_C_) and [C] = 100 × *S*_C_/(*S*_O_ + *S*_C_). The RSFs for the oxygen and carbon core levels were 2.88 and 1.00, respectively, according to Khervefitting software.^45^ The oxygen-to-carbon ratio was defined as [O]/[C].

All data processing and graph plotting for the characterization techniques were performed by using Origin software (OriginLab Corporation).

## 3 Results and Discussion

### 3.1 Nanoindentation

To identify the mechanical effects caused by different plasma atmospheres and duty cycles (DCs) on the rubber surface, nanoindentation tests were performed. The comparison between the treated and untreated samples is presented in Table 2.

**Table 2:** Surface Contact Stiffness Obtained from Nanoindentation Tests with Treated and Untreated Samples^a^

Sample	Contact stiffness (mN/μm)	Standard deviation (mN/μm)
Untreated	2.24	± 0.06
T1	2.62	± 0.04
T2	2.48	± 0.05
T3	2.50	± 0.02
T4	2.56	± 0.06

a Samples: T1 (30% DC, 20% O_2_); T2 (30% DC, 80% O_2_); T3 (70% DC, 20% O_2_); T4 (70% DC, 80% O_2_).

The nanoindentation tests revealed increased surface contact stiffness in all treated samples compared to untreated NBR. Contact stiffness reflects the material’s ability to store energy during deformation. According to Mahmudzadeh et al., higher stiffness indicates reduced viscous dissipation or plastic deformation due to a denser cross-linked surface network.^47^ Although samples T1 and T4 exhibited the highest stiffness, differences among treated samples were not significant. Variability in average values and standard deviations likely arises from surface roughness, as rubbery materials feature random microstructures, leading to dispersion of nanoindentation results.^48^

The increase in contact stiffness on the treated sample surfaces can be associated with cross-linking reactions on the rubber surface.^9,31,49^ Free radicals formed by chain scission can undergo recombination, unsaturation, branching, or cross-linking, and can also react with oxygen species to form oxidized groups and interconnect rubber chains.^50,51^ According to Resende et al., this process consumes energetic species, promotes the formation of chemical bonds between backbones, and reduces chain mobility.^31^ Higher cross-link density thus enhances the elastic response and stiffness.^6,52–55^ Furthermore, this oxidized layer can hinder oxygen diffusion pathways and improve the barrier properties of the rubber, as reported for plasma-treated polymers.^35,56,57^

Indentations reached a maximum contact depth of 32.2 μm at 50 mN, potentially exceeding the thickness of the oxidized layer. Thus, these results may have been influenced by the underlying untreated rubber material. Assuming the surface treatment operates on the nanoscale, the substantial increase in stiffness observed remains noteworthy. This effect arises because measurements reflect only a small fraction of the treated material. Tifui et al. observed Young’s modulus decay in deep indentations (>60 nm) due to the underlying untreated PDMS.^49^ According to the authors, these results suggest that plasma-modified layer thickness depends on treatment time and parameters.^43,49^

The average roughness (*R*_a_) for both reference and treated samples was 0.261 ± 0.02 μm, indicating no significant difference between them. This value is consistent with NBR and EPDM surface roughness values reported in the literature, which typically vary from 0.1 to 1.0 μm.^16,33,58,59^

### 3.2 Differential Scanning Calorimetry (DSC)

Table 3 shows the effect of different plasma treatments on the *T*_g_ values of untreated and plasma-treated samples, as determined by DSC analysis.

**Table 3:** Effect of Plasma Treatment on the Samples *T*_g_ Evaluated by DSC Analysis^a^

Sample ID	*T*_g_ (K)	Stand. Dev. (K)
Untreated	254.6	± 0.4
T1	254.3	± 0.0
T2	254.2	± 0.7
T3	254.0	± 0.1
T4	254.6	± 0.3

a Samples: T1 (30% DC, 20% O_2_); T2 (30% DC, 80% O_2_); T3 (70% DC, 20% O_2_); T4 (70% DC, 80% O_2_).

DSC results showed no significant variation in *T*_g_, as indicated by the standard deviations. Thus, these results are consistent with the objective of this work, as plasma treatments should provide only surface-specific modification while maintaining bulk properties and integrity.

Low-energy-level plasma treatments typically induce changes in surface free energy, wettability, hardness, and roughness.^11,16,31^ Chaiwong et al. showed that treatment of PLA samples with low pressure and low plasma power (50 W) induced topographical changes, while the melting enthalpy remained unchanged, as determined by DSC analyses.^60^ In contrast, high-voltage treatments can alter polymer crystallinity, melting temperature, and glass-transition temperature, leading to further considerable changes in surface roughness and functionalization.^25,61^ Our profilometry results showed that the surface roughness remained constant even after the plasma treatments. Furthermore, the induced modifications maintained the *T*_*g*_ unchanged, supporting selective surface cross-linking and oxidation as mechanisms capable of hindering gas permeation pathways.

### 3.3 Attenuated Total Reflectance–Fourier Transform Infrared (ATR-FTIR) Spectroscopy

The spectra in Figure 2 show the absorption of chemical groups, allowing a comparison of the effects of plasma treatment, as evaluated by ATR-FTIR analysis. As the ATR-FTIR analysis qualitatively confirms the formation of oxygenated groups on the plasma-treated rubber surfaces, it was applied exclusively to samples T3 and T4 as a preliminary screening to evaluate the extremes of the 70% DC.

**Figure 2:**
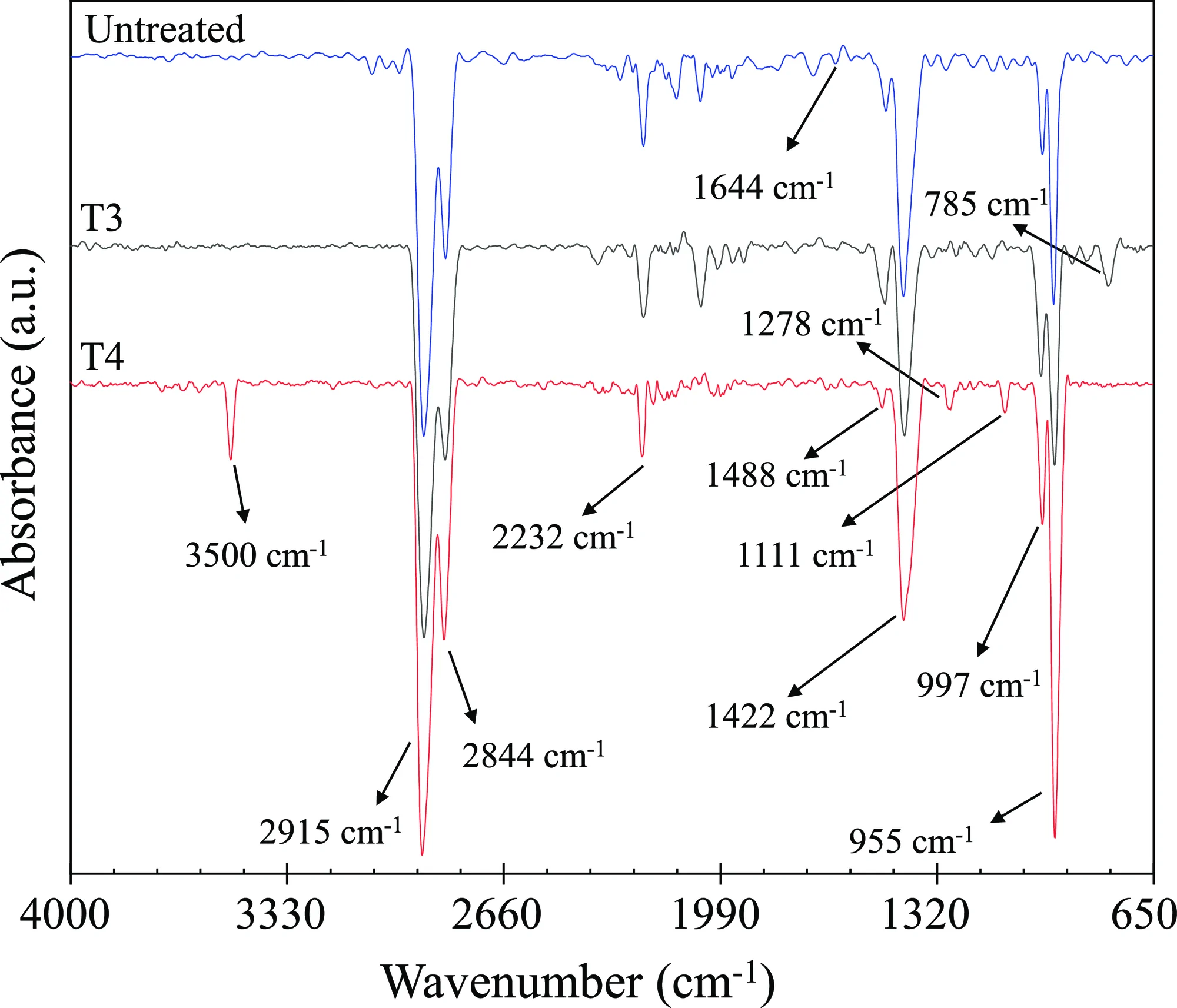
ATR-FTIR spectra for the untreated sample and the samples treated with a 70% duty cycle (DC) with low (T3) and high (T4) oxygen content in the plasma atmosphere. Samples: T3 (70% DC, 20% O_2_); T4 (70% DC, 80% O_2_).

ATR-FTIR analysis of NBR showed the typical groups present in the rubber samples. These groups are represented by bands within the wavenumber range of between 4000 and 650 cm^–1^, as shown in Table 4.

**Table 4:** Typical Peaks Identification in ATR-FTIR Analysis with NBR Samples

Wavenumber (cm^–1^)	Peak assignment
2915 and 2844	–CH_2_, asymmetric and symmetric stretching^6,62,63^
2232	C–N, stretching^63–66^
2050–2290	C–N, other vibrational modes^67^
1644	C=C, stretching^62,66^ or oxidized groups^9,68^
1488	–CH, stretching^69^
1422	CH_2_, deformation^62,69,70^
997 and 955	Silica, C–H or C=C group^62–64,66^

Some absorption bands appeared after plasma treatments in sample T3. The band at 785 cm^–1^ can be assigned to C=C from cis-1,4 butadiene or to an amine group from the phenylenediamine additive;^71,72^ the band at 1111 cm^–1^ can be attributed to C–N stretching of an amine group,^73^ as well as to aliphatic ether (C–O) or alkane (C–H) vibrations;^74^ and the band at 1278 cm^–1^ may be associated with oxidation by subproducts, such as alcohols, ethers, carboxylic acids, or esters,^72^ although these groups are commonly observed in the 1600–1800 cm^–1^ region.^68^ Sample T3 also showed a reduction in band intensity at 1422 cm^–1^ and 2232 cm^–1^. In the spectrum of sample T4, a new band appeared at 3500 cm^–1^, which can be related to −OH groups;^9,72^ the band at 2232 cm^–1^ showed reduced intensity; and bands located at 1111 cm^–1^ and 1278 cm^–1^ also appeared.

Although ATR-FTIR analysis has a relatively high penetration depth,^19,49^ the results for treated samples showed oxygen incorporation (C–O, C=O, O–H) and possibly degradation or removal of side groups, such as CH_2_. These findings directly corroborate the increase in surface contact stiffness, indicating an oxidative effect associated with the formation of a stiffer surface layer (Table 2). Because the micrometer-scale sampling depth of this technique inherently averages the signal from both the modified layer and the underlying bulk, the subtle variations between the 30% and 70% duty cycles could not be significantly resolved. Consequently, high-resolution surface characterization via XPS was prioritized to accurately delineate these nanometric chemical variations, as discussed in subsequent sections.

### 3.4 Optical Emission Spectroscopy (OES)

The emission spectra obtained during the different plasma treatments were collected by using OES, as shown in Figure 3.

**Figure 3:**
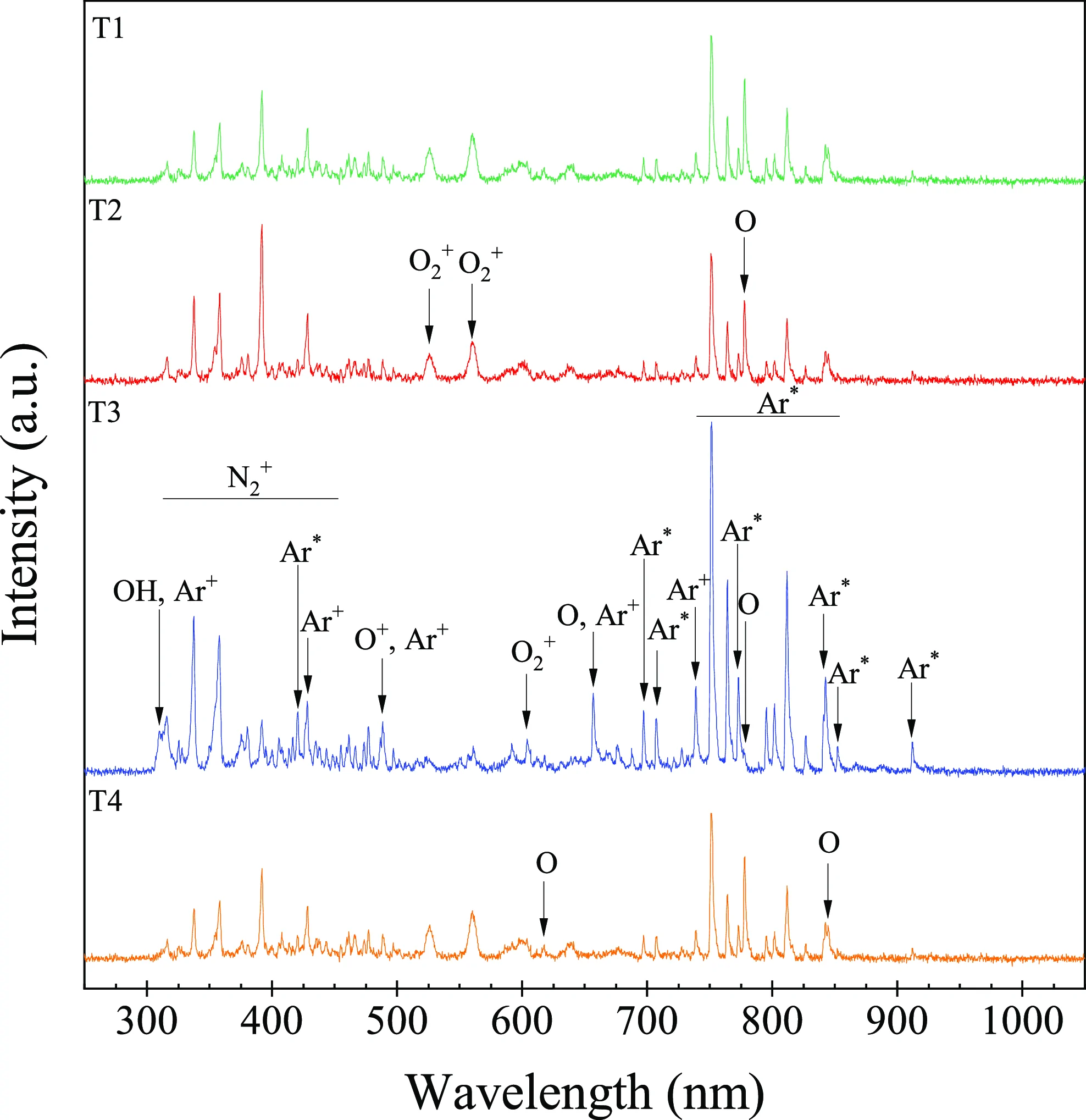
Optical emission spectra of the plasmas used in the treatment of the samples. Samples: T1 (30% DC, 20% O_2_); T2 (30% DC, 80% O_2_); T3 (70% DC, 20% O_2_); and T4 (70% DC, 80% O_2_).

Under all plasma treatment conditions, species related to nitrogen (N_2_), oxygen (O and O_2_), and argon (Ar) were identified. N_2_^+^, O_2_^+^, and Ar^+^ species were associated with positive ions, while O* and Ar* were related to excited species.^75^ Despite the evacuation and purge procedures, the signals for N_2_ species (from 320 to 450 nm)^76^ may be justified by the difference between the base and working pressures (an order of magnitude), which impacts the N_2_ signal.

In general, the treatments generated emission lines from excited and charged argon species (in the 730–850 nm range). Further Ar* lines were identified, with stronger signals in condition T3 compared to the others, at 420.8 nm,^77^ 697 nm,^78^ 707 nm,^77^ 852 nm,^75,76^ and 912 nm.^79^ The main oxygen (atomic oxygen) emission lines were detected under all conditions, which were found at 617.9 nm,^80^ 777.9 nm,^75,81^ and 844.7 nm.^75,81^

Broader peaks centered at 526.4 and 560.1 nm can be associated with excited molecular oxygen (O_2_^+^)^80,82^ and are more evident under conditions T1, T2, and T3. The signal identified at 309 nm may be attributed to the Ar^+^ or OH emission line.^83,84^ The asymmetric signal peaks at 656.7 and 661.3 nm were associated with O and Ar^+^,^79^ respectively. The emission signal at 428.2 nm was attributed to Ar^+^,^79^ and the asymmetric lines at 488.6 nm were assigned to O^+^ and Ar^+^.^79,80^

According to optical emission results, atomic oxygen was generated under all conditions. Higher duty-cycle treatments exhibit a dominant presence of Ar^+^ and Ar* species, which may suppress the emission of O species through competitive excitation pathways while enhancing sample sputtering relative to oxidation. In contrast, lower duty-cycle treatments enhance and favor the generation of the main oxygen species, such as the O and O_2_^+^ lines, which are highly reactive and fundamental to this study’s objectives. This indicates that while oxygen concentrations vary within the plasma volume, their optical emissions are strongly modulated by the energy distribution governed by the argon species.

### 3.5 X-ray Photoelectron Spectroscopy (XPS)

The survey spectra obtained from the wide-scan analyses of untreated and treated samples are presented in Figure 4.

**Figure 4:**
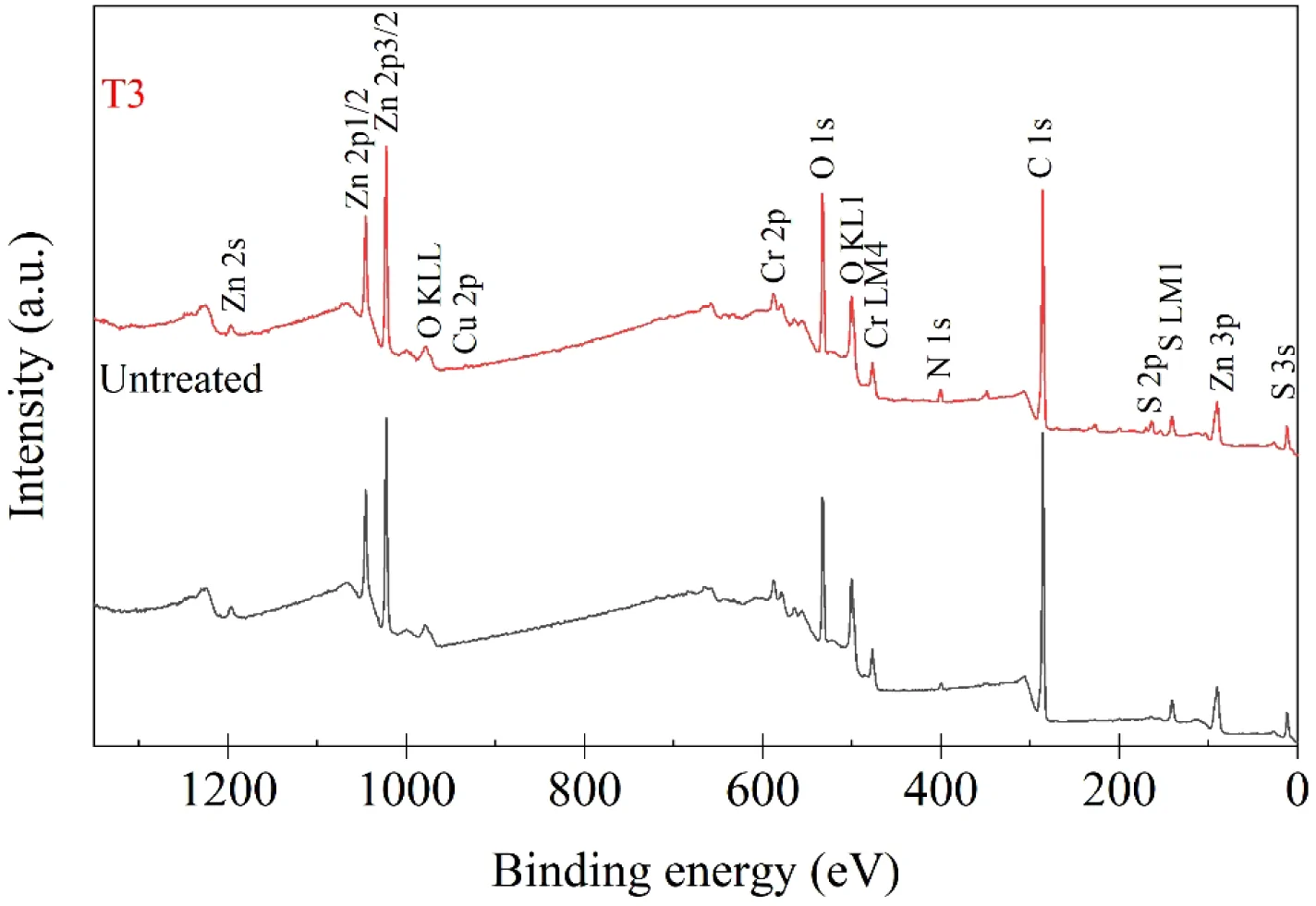
Survey spectra from XPS analyses in untreated and treated samples under different plasma conditions. Sample: T3 (70% DC, 20% O_2_).

The results showed some expected signals related to the butadiene (C 1s) and acrylonitrile (C 1s and N 1s) segments. Further known elements were detected, such as sulfur signals (S 2p, S 3s, and S LM1), associated with the curing agent, or zinc signals (Zn 2p, Zn 2s, and Zn 3p), usually related to ZnO used as a curing activator. The oxygen signal (O 1s) may result from oxidation processes during rubber curing or plasma treatment, including the Auger peaks (O KLL and O KL1). Other specific elements were identified in small quantities, such as Cr 2p and Cr LM4, with the appearance of copper (Cu 2p) in the treated samples possibly associated with sputtering of the substrate holder during the plasma treatment. However, these orbitals were not considered, as they are not expected to significantly influence the material properties.^38^

The regions of C 1s and O 1s core-levels were integrated and quantified by employing high-resolution scans at the surface (cycle 0) for the untreated sample and the sample treated under the T3 condition (70% duty cycle and 20% O_2_/80% Ar), as shown in Figure 5.

**Figure 5:**
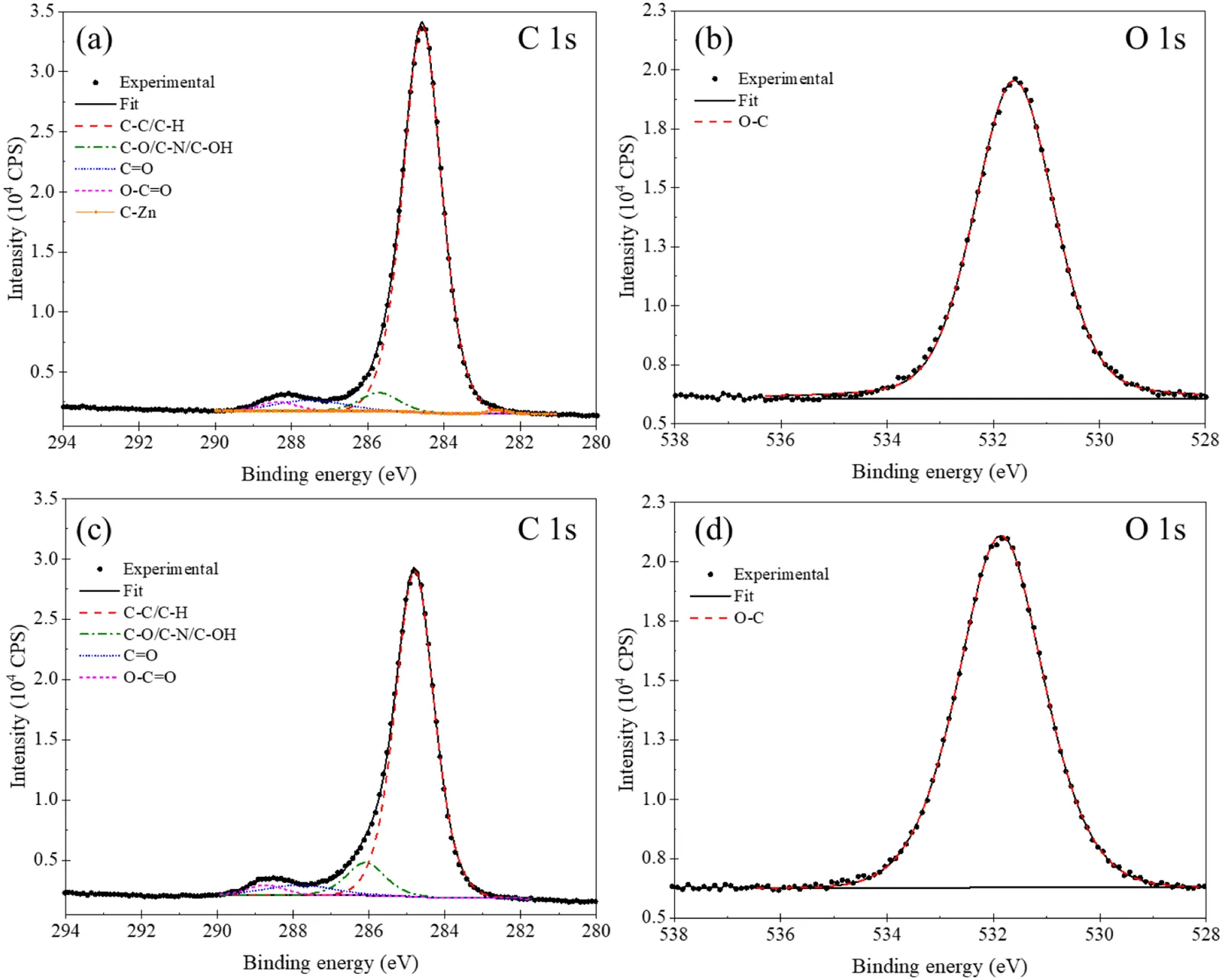
High-resolution XPS spectra of the C 1s (a, c) and O 1s (b, d) core-level regions obtained from the surface of the untreated sample (a, b) and the sample treated under condition T3 (c, d). Sample: T3 (70% DC, 20% O_2_).

The spectral fitting and calculated areas for the C 1s core level (Figure 5a and Figure 5c) were performed by considering contributions from different components. The binding energy correction was performed by shifting the peaks to lower binding energies until the reference value for the C–C/C–H peak was reached (284.8 eV).^46^ The reference peak C–C/C–H (peak 1) was associated with the aliphatic carbon or carbon black; the peak located around 286.0 eV (peak 2) can be associated with C–O/C–O-C groups formed by oxidation,^46,62,85^ C–N from the cyano groups,^16,86^ or C–OH (hydroxyl groups).^46,62^ The peaks at 287.7 eV (peak 3) and 288.6 eV (peak 4) were assigned to C=O^85^ and O–C=O,^62^ respectively, and are related to sample oxidation.

The overall analyses showed that the carbon level (C 1s) tended to decrease, while the oxygen level (O 1s) tended to increase as a result of plasma treatment. For the untreated sample, the main peak (peak 1) comprises 89.0% of the C 1s core-level composition, while for the sample treated under T3 conditions, this peak accounts for 84.4% of C 1s.

The contribution of peak 2 in the C 1s core-level spectrum found in the untreated sample, which corresponds to 4.3% of the C 1s area, can be attributed to C-N (cyano group) from the acrylonitrile molecule or oxidation (C–O or C–OH) from the rubber curing process and unavoidable side reactions during cross-linking with sulfur and other agents,^62^ which involves elevated temperature.^87,88^ However, the C-N bond from the NBR structure was not considered due to nitrogen’s small contribution.^89^ In the treated sample, the contribution of peak 2 in the C 1s core level increases to approximately 8.5% in comparison to the untreated sample. The higher contribution of peak 2 can be associated with the incorporation of oxidized species related to the curing process during rubber manufacturing (elevated temperature),^87,88^ reaction of these species with residual curing agent (reactivation by plasma treatment), or even a direct plasma influence (active site creation and oxidation). Due to the increase in surface contact stiffness (Table 2), the increase in peak 2 can be associated with an increase in cross-links between rubber chains caused by oxidation.^6,32,54^

The contribution of peak 3 to the C 1s core-level in the untreated sample was 4.4%, while in the T3 sample it was 4.6%. The influence of peak 4 in the C 1s core-level area for the untreated sample was 1.9%, whereas for sample T3 it was 2.5%. Moreover, the untreated surface revealed a signal related to a carbide (C-metal bond),^90^ with a binding energy (∼282.6 eV) lower than that of the reference carbon (C–C),^91,92^ which can be associated with the Zn signal observed in the survey analysis (Figure 4).

Regarding the O 1s core-level spectra, Figure 5b and Figure 5d show an increase in peak area after plasma treatment, indicating oxygen incorporation at the rubber surface. The O 1s peaks are located near 531.7 eV, considering the spectral shift in binding energy referencing. Peak symmetry can vary with treatments; narrowed peaks can indicate the influence of a specific bond, *e*.*g*., C–O, while broader peaks can be associated with the influence of both C–O and C=O bonds in the oxidized layer. According to Resende et al., the abstraction of C–H bonds and the formation of oxidized species (C–O and C=O bonds) lead to the formation of a more functionalized surface.^31^ Moreover, some authors also identified the incorporation of hydroxyl groups (OH) in XPS analysis at binding energies around 531.0 eV.^93,94^

According to Resende et al., the decrease in C 1s peak intensities is not due to carbon species removed from the rubber chain, but rather to the incorporation of oxygen species (detectable by XPS) into the rubber chains, as well as the abstraction of hydrogen by oxygen species, thereby generating new oxidized groups.^31^ Furthermore, these results also corroborate those obtained for NBR samples treated with atmospheric-pressure air plasma, in which the plasma-treated samples showed a higher presence of oxygen and a lower presence of carbon at their surfaces than the untreated NBR substrates, representing surface oxidation.^10^

Although the outermost surface layer was oxidized during treatment, it is essential to evaluate the oxygen implantation throughout the sample thickness. On the basis of the evaluation of the C 1s and O 1s core-level spectra, considering their respective relative sensitivity factors (RSFs), the concentrations of C 1s and O 1s on the surface were determined as a function of the sputtering cycle in XPS depth-profile analysis, as shown in Figure 6.

**Figure 6:**
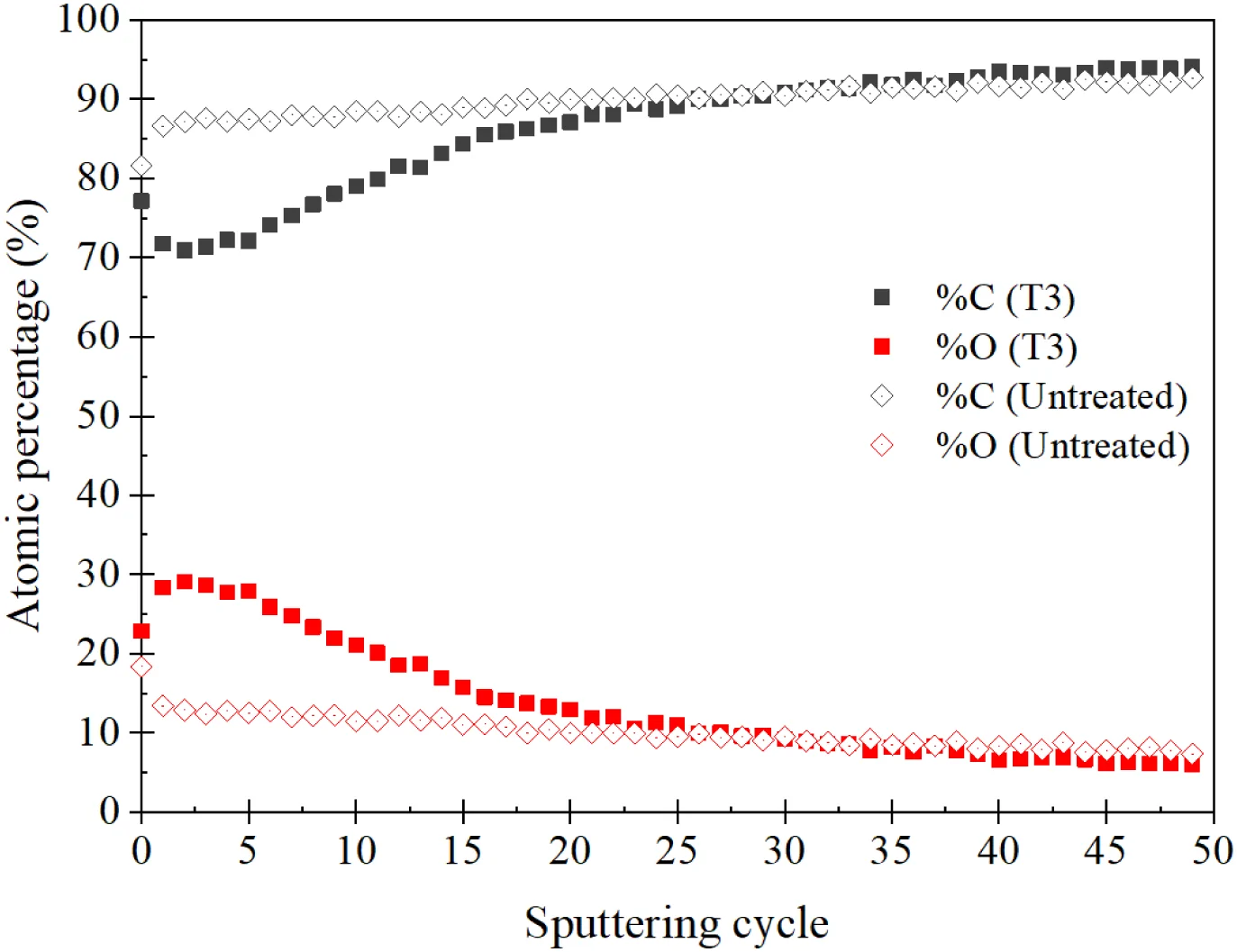
Atomic percentages of C 1s and O 1s core levels from high-resolution XPS with depth-profile analyses. Sample: T3 (70% DC, 20% O_2_).

The result shown in Figure 6 reinforces the oxidation of the outer surface layer: after an initial rise in the oxidation profile was observed in earlier sputtering cycles, it decreased to the same levels as in higher sputtering cycles for untreated samples. This oxidation profile can support nanoindentation results, in which the treated samples exhibited higher surface contact stiffness than the untreated sample (Table 2). At the same time, the bulk properties remained unchanged, as demonstrated by the DSC results (Table 3). Thus, considering the sample characterizations and what occurred with T3 samples, the other treated samples should exhibit higher oxidation levels than the untreated sample due to oxygen implantation during the plasma treatment.^31,38^

The oxygen implantation profile was also evaluated using the SRIM software^95^ by modeling an amorphous layer (20 nm thick) with an approximate density of 1.35 g/cm,^3,96^ and a chemical composition consistent with that obtained from the wide-scan XPS spectrum of the untreated sample (Figure 4). The simulation considered the normal incidence of 10^5^ oxygen atoms onto the NBR surface with a kinetic energy of 600 eV, comparable to the voltage drop in the negative sheath of the substrate holder.

Figure 7 shows an implantation profile consistent with the XPS results (Figure 6), with an average depth of ∼5.9 nm (projected range, *R*_p_) and implanted atoms reaching depths of up to ∼20 nm, indicating that the oxygen incorporation is predominantly confined to the near-surface region.

**Figure 7:**
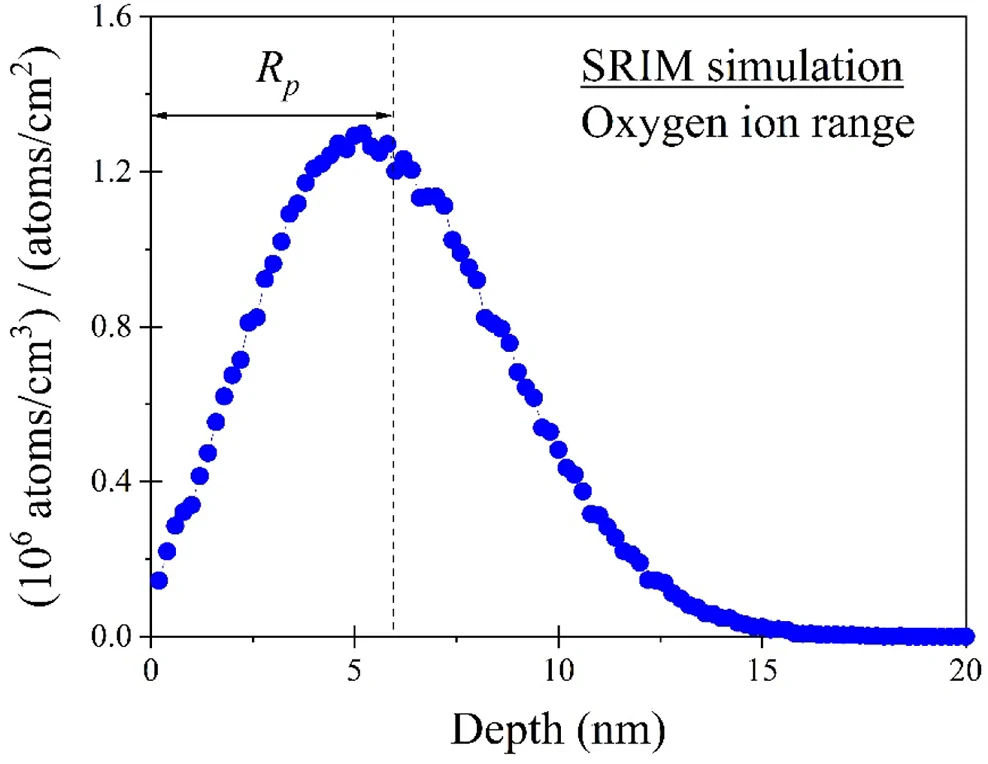
SRIM-simulated depth profile of oxygen implantation.

The deconvolution of the C 1s core level along the depth profile was performed with the untreated and T3 samples, as shown in Figure 8.

**Figure 8:**
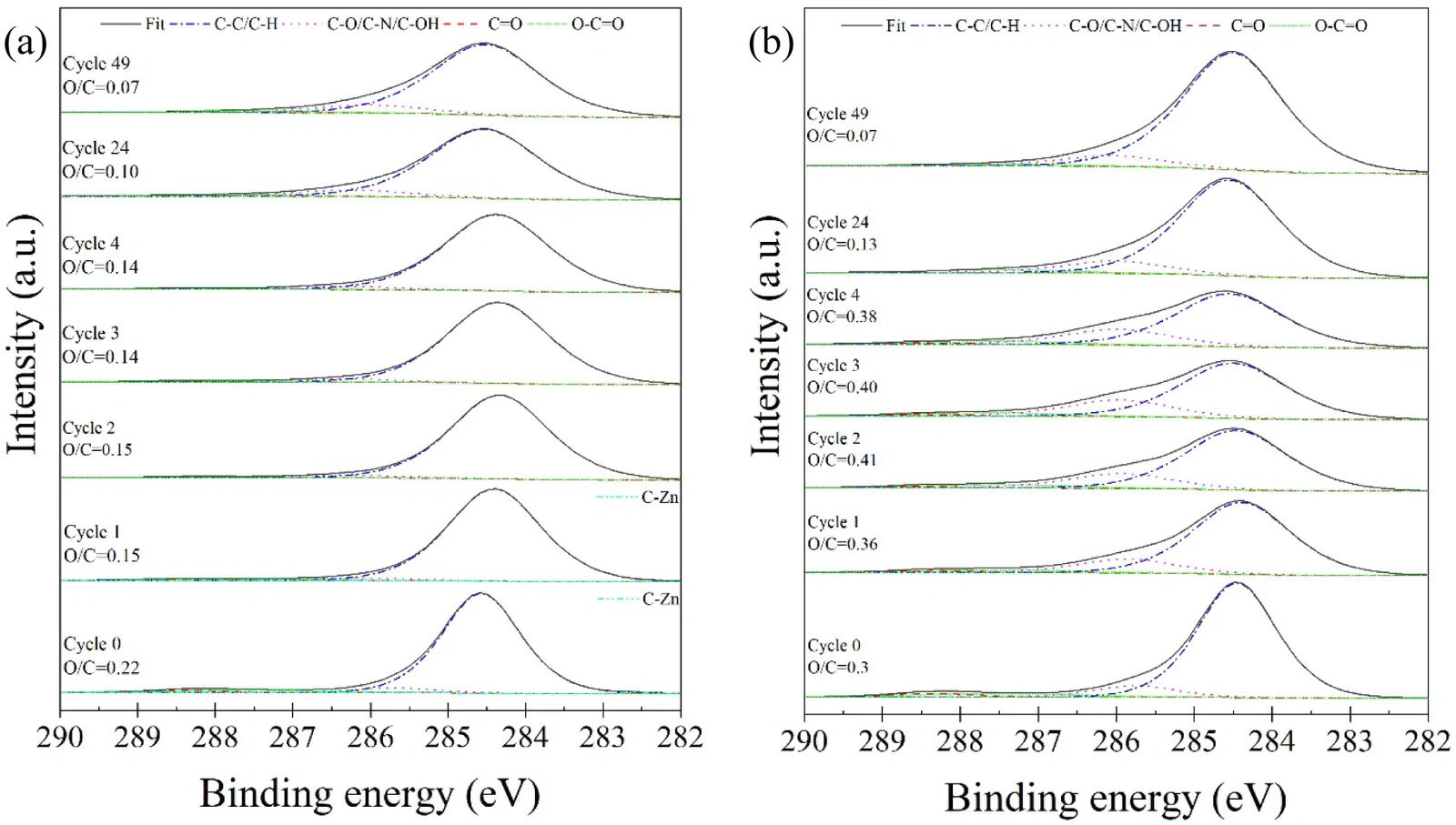
XPS depth profiles showing C 1s core-level deconvolution and O/C ratio as a function of sputtering in the samples (a) untreated and (b) T3. Sample: T3 (70% DC, 20% O_2_).

These results were consistent with those of Shen et al.,^16^ who showed that after exposure to plasma, the percentage of oxidized groups increased with processing time up to 10 min, which was the condition with the highest concentration of oxygen species.^16^ Martínez et al. observed an increase in the O/C ratio from 0.129 to 0.351 for NR-SBR treated with atmospheric-pressure plasma.^9^

The untreated sample showed a peak around 282.5 eV in the first two sputtering cycles, which may be associated with zinc (C–Zn) due to the notable presence of this element in the rubber, as shown in the survey analysis (Figure 4). On the basis of the atomic percentages of carbon and oxygen (Figure 6) and the computed O/C ratio (Figure 8) from XPS depth profile analyses, oxygen implantation and oxidation can be confirmed at the treated sample surface. Along the sample thickness, a higher oxygen concentration is observed in sample T3 (Figure 8b) compared with the untreated sample (Figure 8a). A contrasting behavior was observed when comparing the two conditions: the untreated sample showed an initial decrease in oxidation level from the surface, whereas sample T3 showed an initial increase from sputtering cycle 0 (surface) to cycle 2, which corroborates the aim of creating an oxidized layer on the rubber surface. This result is consistent with the appearance of new bands in ATR-FTIR analyses corresponding to vibrations of oxidized groups on the rubber surface (Figure 2). In the deep region (sputtering cycle 49), the same oxidation level (O/C ratio) is observed for both sample conditions, substantiating the objective of unchanged bulk properties, as shown by the DSC results (Table 3).

### 3.6 Plasma–Surface Interaction

When the rubber specimens were subjected to Ar/O_2_ plasma treatments, modifications occurred due to energetic species bombardment onto the rubber surface. The collisions of high-energy particles with the rubber surface break weaker bonds in the polymer chains (mainly C = C and C–H), while backbone C–C and C-N remain stable due to their higher resistance to direct breakage.^97^ These collisions lead to the formation of free radicals on the polymer surface,^38,97^ where many reactions (recombination, unsaturation, branching, and cross-linking) can occur in order to provide surface functionalization by the presence of highly reactive oxygen species in the plasma atmosphere.^25,38,98^ Thus, highly reactive oxygen species rapidly interact with active sites on the polymer surface via chemisorption,^8^ as shown in the proposed reaction mechanism in Figure 9.

**Figure 9:**
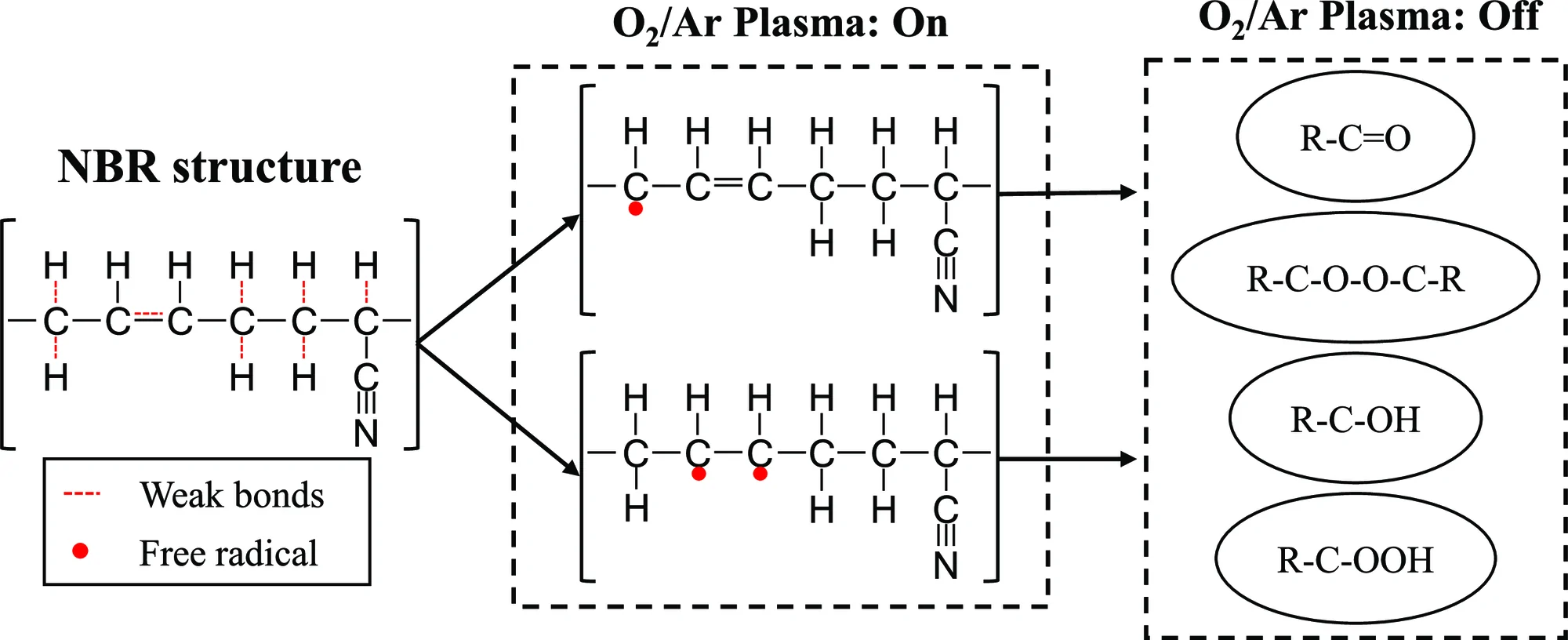
Mechanism for the main oxidation reactions during pulsed Ar/O_2_ plasma in NBR.

According to the nanoindentation results (Section 3.1), the oxidative reactions near the rubber surface appear to be dominated by cross-linking, as the contact stiffness of treated samples increases relative to that of the untreated sample. It can be divided into H abstraction for C–H bonds, mainly in secondary and tertiary carbon, and saturation of C=C bonds, due to their weaker bond energies.^6^ Moreover, the oxygenated groups on the rubber surface can be classified as lateral/chain-end groups (R–C=O, R–COH, and R–COOH) and chain cross-links (R–C–O–O–C–R), where an increase in cross-link increases result in increased surface stiffness (see Table 2). This also corroborates the XPS results, in which analyses of treated samples showed a greater influence of surface-oxidized groups compared to the untreated sample (Figures 5) and across the sample thickness (Figures 6 and 8), as also demonstrated by the simulation of oxygen implantation depth (Figure 7).

During pulsed-plasma treatments, the duty cycle directly affects the sample exposure time, the temperature increase during treatment, and effective bombardment events, as the pulse-on accelerates energetic positive ions to the substrate surface, while the pulse-off neutralizes the surface, allowing chemical interaction with the atmosphere.^27^

Figure 10 presents a scheme for the physical and chemical effects as functions of the duty cycle.

**Figure 10:**
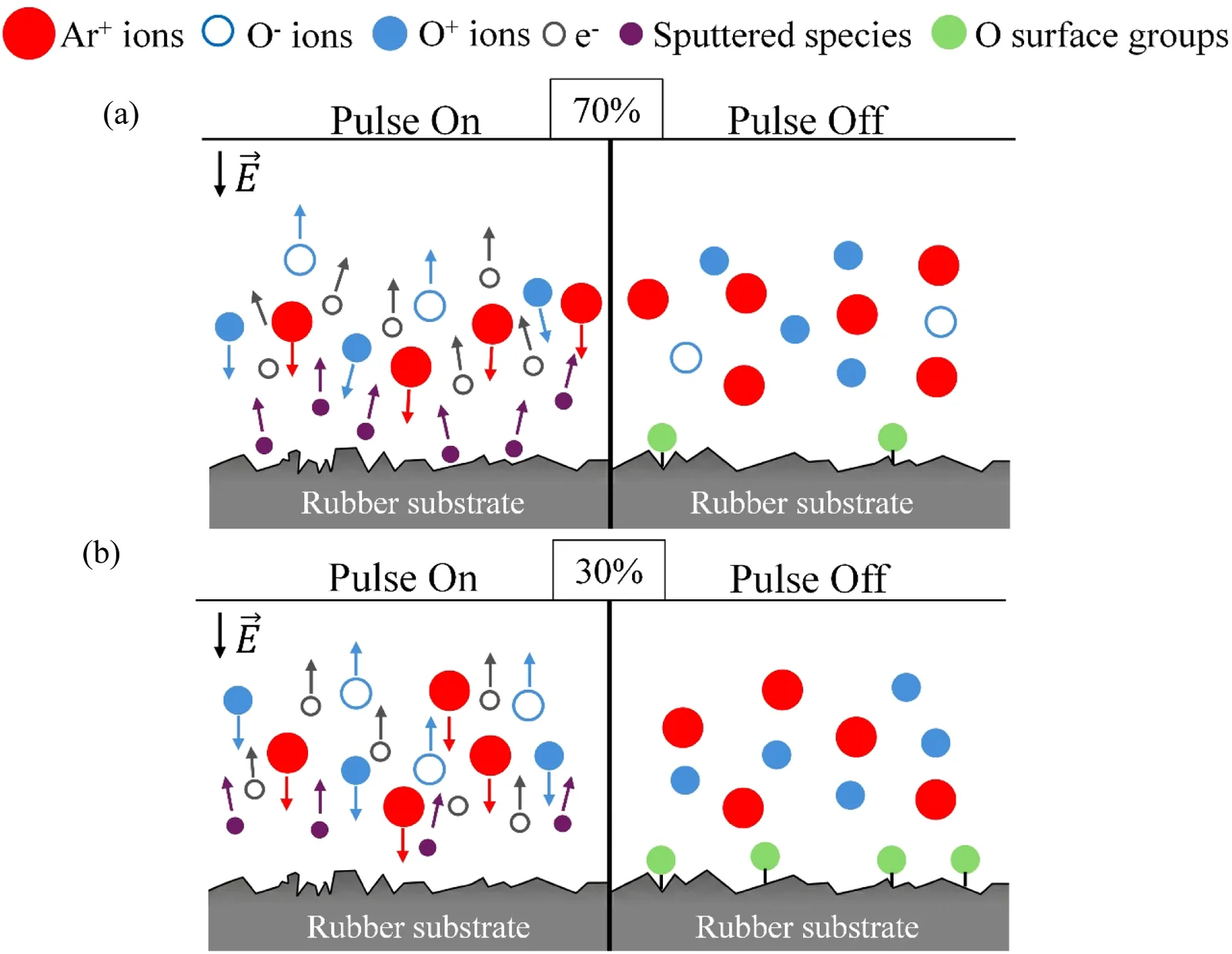
Proposed mechanisms for plasma–surface interactions with NBR samples during pulsed plasma at: (a) 70% duty cycle and (b) 30% duty cycle.

The higher duty cycle level is responsible for the bombardment of ions for 70% of the treatment time, which promotes physical sputtering of species with weaker bond energies and restricts the surface–atmosphere reaction to only 30% of the time. Conversely, the lower duty cycle is more susceptible to producing complete surface reactions due to extended pulse-off periods. With a 70% duty cycle (Figure 10a), more sputtered species and fewer oxidized species are present on the substrate surface than at a 30% duty cycle (Figure 10b).^19^ Higher duty cycles also produced more energetic species, as presented by OES results (Figure 3), which increases the sputtering level, especially in T3 and T4 treatment conditions, where various energetic species can be observed.

The gas composition during plasma treatment also influences the rubber surface modification: inert Ar^+^ ions can efficiently generate new free radicals via physical sputtering of surface species (C=C and/or C–H), while highly reactive oxygen species (O, O^+^, and O_2_^+^) oxidize these new radicals.^19^ Thus, higher oxygen content (T2 and T4) enhances the plasma reactivity and the atomic oxygen flux in comparison to lower oxygen content (T1 and T3),^26^ as evidenced by OES (Figure 3) and ATR-FTIR results (Section 3.3). This can also be observed in the XPS and O/C ratio results (Figures 5 and 8) when comparing the untreated and treated (T3) samples. However, when using a gas mixture with 20% O_2_, more Ar^+^ ions were generated (Figure 3), thereby boosting the sputtering efficiency and increasing active site formation relative to oxidation.^26^ Thus, among the evaluated duty cycles and gas mixtures, treatment T2 is expected to yield the highest degree of oxidation, owing to the lowest sputtering rates and the highest concentration of oxygen species in the plasma. This condition is well-suited to generating an oxidized surface barrier that inhibits gas permeation during operation, particularly in thermooxidative environments.

## 4 Conclusions

In this work, NBR samples were subjected to a surface treatment using a low-pressure pulsed plasma with two different duty cycles (30% and 70%) and two different plasma atmospheres (20%/80% O_2_/Ar and 80%/20% O_2_/Ar).

The surface contact stiffness increases across all treatment conditions, as verified by nanoindentation tests, and can be directly attributed to peroxy links between rubber chains. The samples’ thermal behavior was assessed using DSC to determine the glass transition temperature. This result showed that the bulk properties remained unchanged after the adopted plasma treatments.

Chemical analysis was carried out by using ATR-FTIR and XPS. FTIR results exhibited new bands that can be associated with vibrational modes of oxidized groups, as well as reduced hydrocarbon bands, suggesting oxidation. XPS analyses confirmed the introduction of oxidized groups onto the rubber surface by the presence of specific bonds in the high-resolution C 1s and O 1s core levels, such as C–O/C–OH, C=O, and O–C=O. The treatment increased the contribution of oxidized components in the C 1s high-resolution core-level spectrum, as well as the O 1s area and the O/C ratio, compared with the untreated sample, supporting an increase in contact stiffness due to oxidative cross-links. Depth-profile analyses revealed elevated surface oxidation in the T3-treated sample, characterized by an initial increase followed by a gradual decrease in atomic oxygen concentration with depth (*i*.*e*., increasing sputtering cycles), reaching a depth of up to 20 nm, as observed in oxygen-implantation simulation. In contrast, the untreated sample exhibited a monotonic decrease in oxidation throughout the profile.

A plasma-surface mechanism proposes oxidation via H-atom abstraction from C–H bonds or saturation of C=C bonds, followed by oxidation by highly reactive plasma species, such as atomic oxygen. A higher duty cycle (70%) implies higher sputtering levels with a dominant destruction of weaker surface species over oxidation processes, whereas a lower duty cycle (30%) allows more time for oxygen implantation. These effects are amplified by variation in the Ar/O_2_ gas mixture: oxidation processes scale with the O_2_ content, while sputtering escalates with Ar, as observed in surface XPS results. OES results showed stronger emission lines for Ar species for the highest duty cycle, while the highest oxygen content was more prone to the formation of O species.

The treatment T2 (30% DC, 80% O_2_, and 20% Ar) potentially yielded a higher surface oxidation level, an ideal condition for forming a robust surface barrier and blocking oxygen under operating conditions, thereby enhancing durability through enhanced oxidation resistance. Thus, Ar/O_2_ pulsed plasma offers a suitable surface treatment for enhancing NBR, targeting barrier properties, and improving surface oxidation cross-linking and stiffness by employing precise control of the duty cycle and gas mixture within the specific operational windows investigated in this work.
